# Comparative epigenomics by machine learning approach for neuroblastoma

**DOI:** 10.1186/s12864-022-09061-y

**Published:** 2022-12-27

**Authors:** Ryuichi P. Sugino, Miki Ohira, Sayaka P. Mansai, Takehiko Kamijo

**Affiliations:** 1 Research Institute for Clinical Oncology, Saitama Cancer Center, Ina, Saitama, 362-0806 Japan; 2 Laboratory of Tumor Molecular Biology, Department of Graduate School of Science and Engineering, Saitama University, Kita-Urawa, Saitama, Japan

**Keywords:** Comparative epigenomics, Machine learning, DNA methylation, Neuroblastoma

## Abstract

**Background:**

Neuroblastoma (NB) is the second most common pediatric solid tumor. Because the number of genetic mutations found in tumors are small, even in some patients with unfavorable NB, epigenetic variation is expected to play an important role in NB progression. DNA methylation is a major epigenetic mechanism, and its relationship with NB prognosis has been a concern. One limitation with the analysis of variation in DNA methylation is the lack of a suitable analytical model. Therefore, in this study, we performed a random forest (RF) analysis of the DNA methylome data of NB from multiple databases.

**Results:**

RF is a popular machine learning model owing to its simplicity, intuitiveness, and computational cost. RF analysis identified novel intermediate-risk patient groups with characteristic DNA methylation patterns within the low-risk group. Feature selection analysis based on probe annotation revealed that enhancer-annotated regions had strong predictive power, particularly for MYCN-amplified NBs. We developed a gene-based analytical model to identify candidate genes related to disease progression, such as *PRDM8* and *FAM13A-AS1*. RF analysis revealed sufficient predictive power compared to other machine learning models.

**Conclusions:**

RF is a useful tool for DNA methylome analysis in cancer epigenetic studies, and has potential to identify a novel cancer-related genes.

**Supplementary Information:**

The online version contains supplementary material available at 10.1186/s12864-022-09061-y.

## Background

Neuroblastoma (NB) is the second most common pediatric solid tumor [1, 2], and its risk has been classified by a variety of clinical and biological markers. Tumor stage is determined by the International Neuroblastoma Staging System (INSS) [3, 4], and reflects the prognosis of NB patients [5]. Amplification of *the MYCN* gene is one of the strongest prognostic markers for NB [6–8]. Age at onset is also a strong marker of prognosis, and patients under 18 months of age tend to have a favorable prognosis [9, 10]. The International Neuroblastoma Risk Group (INRG) classification system merges information including stage, age at onset, DNA ploidy, pathology, and *MYCN* status for prognosis [11]. Some gene statuses, including *ATRX* gene deficiency and *TERT* gene rearrangement [12, 13], have been reported to be related to unfavorable prognosis; however, these genomic variations have been detected in fewer than half of the patients with unfavorable NB.

In NB, epigenetic alterations influence NB tumorigenesis and aggressiveness. DNA methylation, a major regulator of gene expression, of tumors is considered a prognostic marker for NB. The CpG island methylation phenotype (CIMP) marker of NB was first studied using cell line DNA, and its prognostic impact was confirmed using clinical samples [14–18]. Additionally, Genome-wide DNA methylome analysis also showed that DNA methylation status is strongly related to NB prognosis. Decock et al. [19] selected 43 candidate markers from the methylome data of 5-aza-2′-deoxycytidine (DAC) treatment and MBD-seq analysis, and found a relationship between DNA methylation and risk factors such as age, stage, and *MYCN* amplification. Comparative DNA methylome analysis of clinical samples showed that variable DNA methylation sites were observed on the gene body and within the intragenic regions rather than the “promoter region” [20, 21] of a gene, and some prognosis marker genes, such as *CCND*, were proposed. Henrich et al. [22] showed that the DNA methylation pattern is related to NB status, specifically *MYCN* amplification. To date, genome-wide DNA methylome data (Illumina humanmethylation 450 K beadchip Array [23]) have been obtained from 493 patients diagnosed with NB, and have three advantages for machine learning applications. First, unlike gene expression data obtained using various platforms, meta-analysis was easy to apply because the data format was consistent. Second, DNA methylome data generally ranged from 0 to 1 when using *β-values*, meaning further normalization was not required. Third, because the machine learning model is a data-driven analytical model, it can be easily applied to multiclass data such as tumor stage.

Notably, the machine learning (ML) approach has been applied to gene expression data in NB to construct a classifier for the prognosis of patients with NB. For example, Ohira et al.selected prognosis-related genes and developed a diagnostic mini-chip system consisting of 200 genes using a supervised machine learning algorithm [24]. Oberthur et al. applied a support vector machine (SVM) to classify high-risk patients using microarray data and proposed a new risk classification system [25, 26]. A combination of biology-driven feature selection and artificial neural network analysis can predict the stages of NB [27–29]. Zhang et al. [30] integrated gene expression data with copy number variation data, analyzed them using machine learning methods, and ultimately developed two classes of high-risk patients with neuroblastoma. Grimes et al. estimated the survival time from a regression analysis of RNA-seq data [31]. Giwa et al. identified *MYCN*-amplified sample-specific DNA methylation sites using comparative DNA methylome analysis including ML [32]. Lalchungnunga et al. used unsupervised machine learning approach and identified low risk tumor group [33]. These approaches suggest that ML have a potential of systematic prediction for NB prognosis.

Here, we applied machine learning analysis to NB DNA methylome data sourced from multiple databases. Because of the lack of an analytical model, only simple statistical tests have been applied to DNA methylome data (e.g., *t*-test analysis for differentiated DNA methylated regions between two classes of neuroblastoma) thus far. To address this statistical power gap, we used random forest (RF) [34] to analyze DNA methylation array data. RF has the advantage of multiclass classification, which is a known characteristic of NB [22]. We found that: (1) novel intermediate-risk patient groups are identified using RF-based multiclass analysis of intermediate risk subgroups using DNA methylation data, (2) enhancer DNA methylation is the best annotation group for predicting *MYCN* status. (3) RF has sufficient power for prognosis, and a longer time course resulted in better prediction. Overall, our analyses revealed that the machine learning model is a strong tool for the analysis of tumor DNA methylation status as an epigenomic biomarker of malignant NB tumors.

## Results

### DNA methylome data from multiple studies

We collected Infinium HumanMethylation450 methylation data from four research projects: TARGET (*n* = 211), Henrich et al. [22] (*n* = 80), Ackermann et al. [35] (*n* = 58), and Japan Childhood Cancer Group Neuroblastoma Committee (JCCG-JNBSG) (JNB, *n* = 144, Ohira M et al.*,* manuscript in preparation) (Table S1). Because the research interests may primarily focus on high-risk patients in these projects, 68% of data collected were from stage IV samples, introducing a bias toward later stages in the datasets.

**Fig. 1:**
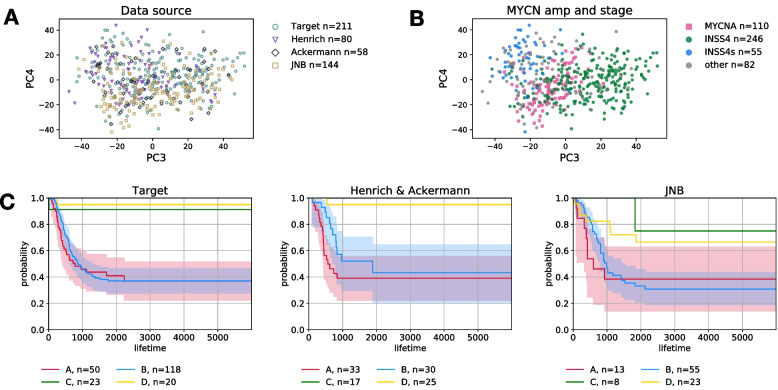
Data comparison among research project on DNA methylome data **(A)** PCA result colored by data source. **(B)** PCA result colored by *MYCN* amplification status and INSS. The definition of group A, B, C, and D is explained in main text. **(C)** Survival time analysis for each dataset

We checked data source bias among the research projects using principal component analysis (PCA) (Fig. 1A and Fig. S1). Further, *β*-values were obtained using the same protocol from deposited idat files (see Materials and Methods). The PCA results indicated that there was no strong bias in the view of the data source (Fig. 1A and Fig. S1A). When the INSS stage was focused (Fig. S1B), stage IV showed a weak, but not apparent cluster. This may be due to the fact that most of the samples were from stage IV cases, or because the DNA methylation pattern of stage IV was variable. In contrast to the INSS stage, *MYCN* amplification showed a distinct cluster (Fig. S1C), indicating that *the MYCN* amplification status affects DNA methylation patterns. Patients under the age of 1.5 years at diagnosis also look weak cluster, indistinguishable from stage IV (Fig. S1D). From these results, we set the following four classes for machine learning analysis (Fig. 1B): Group A, patients with *MYCN* amplification; Group B, INSS stage IV cancer patients without *MYCN* amplification; Group C: INSS stage IVs cancer patients; and Group D: INSS stage I, II, and III patients without *MYCN* amplification. This classification accurately reflects prognosis and confirms consistency among the datasets (Fig. 1C).

### Classification of NB stage by methylome data

To evaluate the adequacy of our classification, we applied RF analysis to 450 K data. To reduce sampling bias, a hold-out train-test-split with 1000 replications was applied. Table 1 shows the results of the prediction scores of the NB methylome dataset. When we focused on the high-risk class (groups A and B), the mean precision (plus standard deviation), the recall for A and B was 0.946 ± 0.039 and 0.798 ± 0.028, 0.855 ± 0.060 and 0.963 ± 0.021 respectively, indicating that high-risk NB is associated with changes in DNA methylation patterns. In contrast, the prediction ability of the low-risk class was not good, indicating that either DNA methylation status was not characteristic, or our classification was inappropriate. When setting stage III as an independent class, the mean precision score was 0.053, while the mean recall score was 0.009. This indicated that stage III was not a characteristic category of DNA methylation status in this dataset. When classification was performed across all datasets, the results were not very different, with the exception of the JNB data (Fig. S2). We also evaluated sampling bias toward high risk patients by controling sample size, and confirmed the result was consistetnt.

**Table 1:** RF result of 1000 replication

	A’	B′	C′	D′	3
n	110	246	55	53	29
precision	0.931 ± 0.036	0.833 ± 0.036	0.577 ± 0.118	0.414 ± 0.127	0.053 ± 0.209
recall	0.881 ± 0.058	0.926 ± 0.030	0.730 ± 0.119	0.350 ± 0.111	0.009 ± 0.033
f1	0.904 ± 0.034	0.876 ± 0.024	0.633 ± 0.089	0.366 ± 0.093	0.015 ± 0.055

**Fig. 2:**
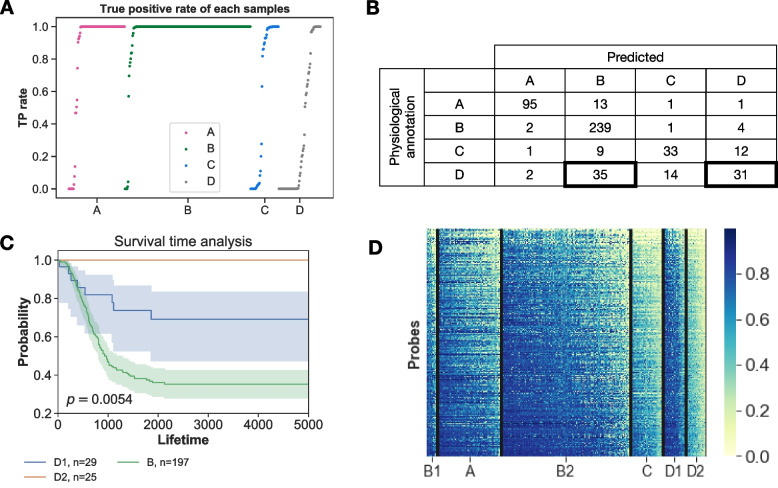
Details of prediction by RF. **(A)** Proportion of TP for each sample. X-axis is samples. Y-axis is TP rate estimated by 1000 times replications. **(B)** Summary of the most assigned class for each sample. **(C)** Survival time analysis for B, D1, and D2. The samples with survival data were used. **(D)** Heatmap for probes with *∆β*_*|D1-D2|*_ > 0.3 (*n* = 3319 probes). X-axis indicated sample class. Group B1 represents INSS 4 without *MYCN* amplification of < 1.5 years old patient group, while B2 is the remaining Group B samples

Compared to groups A and B, the prediction scores were lower for groups C and D (Table 1). To identify the cause of misclassification, we checked the details of the misclassified samples. Specifically, if some samples were imitated to different classes in physiological diagnosis, it would result in a low prediction score. Figure 2A shows the true positive (TP) rate of each sample. Most of the samples from groups A and B were classified accurately. However, only approximately half of the samples from groups C and D were accurately classified. To test the possibility of imitation, we summarized the most assigned group for each sample using the result of the confusion matrix (Fig. 2B). In group D, 35/82 (43%) samples were classified as group B. It can be hypothesized that these samples look physiologically like low-risk patients, but at the DNA methylation level, they were closer to group B. We set new classes: D1 for group D samples predicted as group B (*n* = 35), and D2 for group D samples predicted as group D (*n* = 31) (Fig. 2B, Surrounded by thick frames). Survival time analysis showed a clear discrepancy between D1 and D2 (Fig. 2C, *p* = 0.0054), even though the survival probability in group D was higher than that in group B. Next, we compared DNA methylation patterns among classes (Fig. 2D). The variable region between D1 and D2 was selected (*∆β*_|D1-D2|_ > 0.3, 3319 probes). The results of D2 looked similar to group C, and D1 looked similar to the poor prognosis groups, A and B. In most of the probes, DNA methylation levels were higher in the poor prognosis group D1 (3315/3319 probes in Fig. 2D).

The next question was what types of factors contributed to the differences between D1 and D2. One possibility is the INSS stage, considering that state III was assigned to group D, which could explain the difference. However, when the proportions of stage III in D1 (16/35) and D2 (9/31) were compared using Fisher’s exact test, the difference was not significant (*p* = 0.2073). Next, we focused on the age at onset. Age of onset is an important prognostic marker for NB [9, 10], with 1.5-years-old as an important prognostic marker threshold. When we counted the number of patients under the age of 1.5 years in group D and compared them using Fisher’s exact test, the proportion was significantly different (9/35 for D1 compared to 30/31 for D2, *p* = 0.0148). Therefore age-dependent DNA methylation changes may contribute to poor prognosis.

DNA methylation status changes with age [36]. We investigated whether the difference in DNA methylation with respect to age affected prognosis. We compared *∆β* to the log-rank test’s false discovery rate (LRFDR) for all probes (Fig. S3). LRFDR was used as a proxy for probe contribution to prognosis. The 593 probes satisfied both criteria of *∆β*_*|D1 − D2|*_ > 0.3 and LRFDR < 0.01, as shown in the magenta section in Fig. S3A. If these probes were related to poor prognosis, the DNA methylation pattern of those sites would be similar to poor prognosis groups, such as group B. Further, we established new group B1, which is INSS 4 without *MYCN* amplification for age < 1.5 patient group (group B1 in Fig. 2D). B1 was more similar to D1 than D2, particularly with respect to low LRFDR probes (< 10^− 2^) (Euclidian distance: 0. 0973 for *∆β*_*|B1 − D1|*_ vs 0. 2556 for *∆β*_*|B1 − D2|*_, *p* = 0 permutation test) (Fig. S3B, C). This demonstrated that DNA methylation sites were indicators of poor prognosis regardless of the age of onset.

### Feature selection by probe annotation

**Fig. 3:**
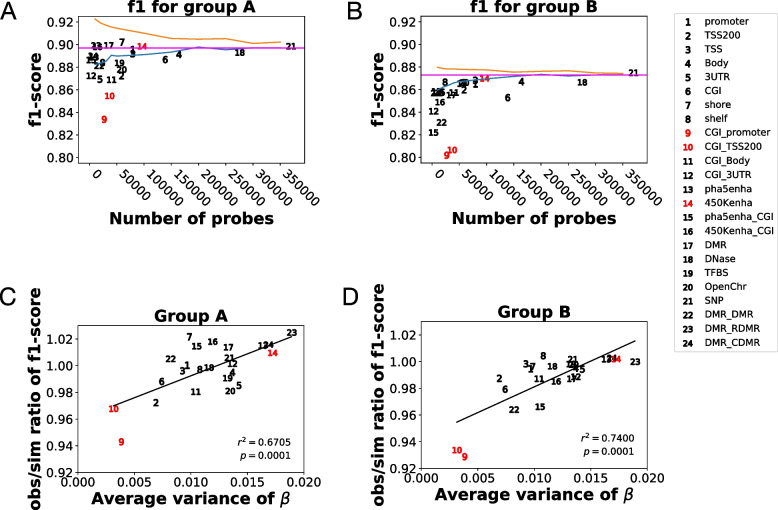
Prediction power of probe groups. **(A)** Effect of probe groups in prediction of group A. Blue line represents the random-sampling of probes. Orange line shows select percentile method of scikit-learn with default setting. **(B)** Group B version of Fig. 3A). **(C)** Relationship between the variance of *β* and *obs/sim* f1-score for group A. **(D)** Relationship between the variance of *β* and *obs/sim* f1-score for group B

Feature selection is a useful approach in machine learning when data are constructed using a large number of variables or expert knowledge is available [37]. Therefore, we applied this method to analyze NB DNA methylation array data, because the sample size (*n* = 493) in the NB DNA methylome data was smaller than the number of variables (*p* > 480,000). Although probes were designed using expert knowledge (e.g., around TSS, CGI, and enhancer), the probe annotation groups that contributed to the classification were unknown. To evaluate the prediction power of the probe annotation groups, we compared the f1-scores of groups A and B, which were calculated using the harmonic mean of precision and recall [37] (Fig. 3A and B and Table S2). Probe annotation groups were defined by EPIC probe annotation (details in Materials and Methods section). We found that Group A was generally accurately classified when “450K_enhancer” probes (probe group 14, Fig. 3A and B) were used, and the enhancer region is known to possess variable *β-values* in NB [20, 21]. Meanwhile, promoter regions with CpG islands had low prediction ability (probe groups 9 and 10, Fig. 3A, B).

We then investigated whether the number of variables was affected by the f1 score. To evaluate the effect, we performed two types of randomization tests: 1) random subsampling of probes (blue line in Fig. 3A and B) and 2) The select-percentile method (orange line in Fig. 3A and B). The select-percentile method was followed by default settings of the scikit-learn library (https://scikit-learn.org/stable/), whose variables were sorted by ANOVA’s resulting *p*-values. Some of the probe groups generally, had better f1-score than those in the random subsampling group, but they always showed worse f1-score than those in the select percentile (between orange line and blue line, in Fig. 3A and B) group.

We then investigated why the CGI and promoter regions showed an unexpectedly low f1 score. We propose that this may be attributed to the variance of the *β*-values. Figure 3C and D illustrate the relationship between variance and the obs/sim ratio, which is the ratio between the f1-score of the observed data and that of the randomly sampled data. The obs/sim ratio of the f1-score was used in classification as the measure of the prediction power of the probe. After performing a permutation test, we observed a significant correlation between variance and the obs/sim ratio of the f1-score (Group A:*r* = 0.6705 and *p* = 0.0001; and Group B: *r* = 0.7400 and *p* = 0.0001.), and found that probes with CGI_promoter and CGI_TSS200 had smaller variances (probe groups 9 and 10, Fig. 3C and D). Those results show that variance of variables is a good indicator for classification power in the case of NB DNA methylation data.

### Random forest selected top-score 10,000 probes for NB classification

Next, we searched for characteristic genes to classify each NB group. To evaluate the classification ability of each DNA methylation site, we used two indices: *importance* and Gini-impurity. *Importance* is the standard index for a decision tree, and it reflects the classification power of each variable [32]. *The importance* was calculated using 1000,000 replications of RF analysis. Gini-impurities are an index of data purification power [37]. We defined the DNA methylation sites of group *k* (∈(A,B,C,D)) (CMS_*k*_) by Gini-impurity, which measured the contribution to classify a class *k* (details in Materials and Methods section). The relationship between the EPIC probe annotation and *importance* is shown in Fig. S4. The probe group with high classification ability would have a negative correlation between the rank of probes based on *the importance* and proportion of focal probes. We found that the probes “450k_Enhancer,” “Phantom5_Enhancer,” and “RDMR” exhibited this pattern (Fig. S4B). When *importance* and CMS_*k*_ were compared, groups A, B, and C exhibited a negative correlation between the rank of *importance* and CMS_*k*_ (top column, Fig. S5), indicating that the classification worked well for those groups. When the threshold for classification (*θ*) was controlled for groups A and B, even low *θ* accurately caught the classification pattern, but for group C, high *θ* was more accurate (top column, group C, *θ* = 0.9). In most cases, the relationship between the rank of *importance* and CMS_*k*_ showed an *L*-shape in groups A and B. These results indicated that a small number of probes contributed to classification, which is consistent with the results of the select percentile patterns shown in Fig. 3A and B. We selected the top 10,000 *importance* probes located on 135 transcripts and 78 gene symbols (Additional file 2 for top 10,000 importance probes and Additional file 3 for 135 transcripts) to confirm the development of the gene-based analytical model and identify candidate genes associated with disease progression.

**Fig. 4:**
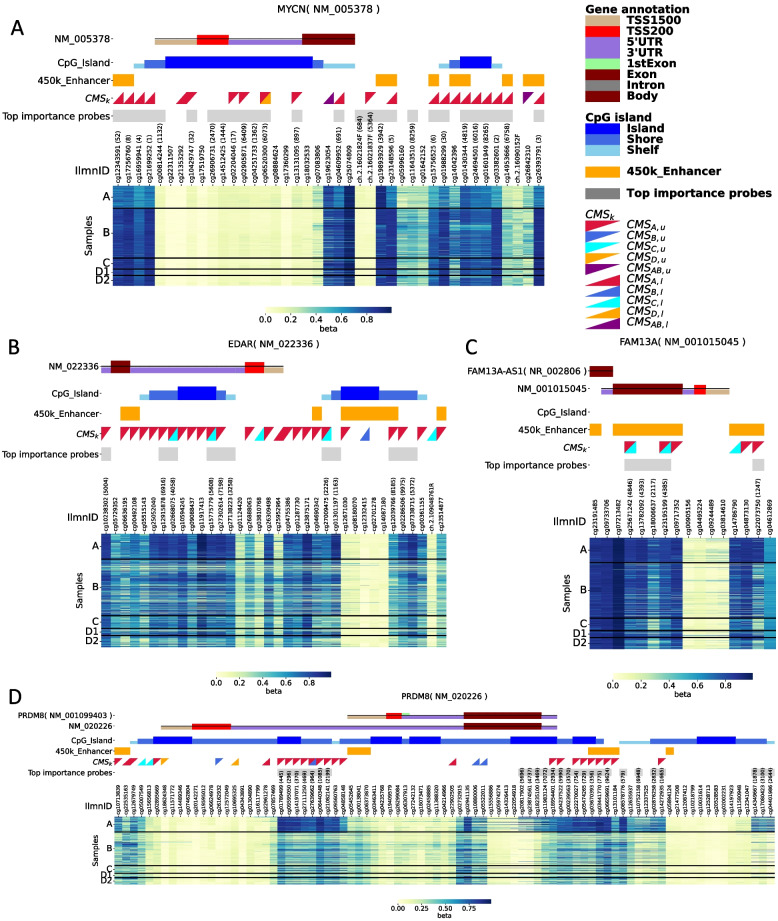
**(A)** DNA methylation pattern of *MYCN* gene (NM_005378) and its neighbor regions at approximately ±100 kb of TSS. Samples were classified as group A, B, C, D1, and D2. From top, annotated genes on refseq (“UCSC_RefGene_Group”), relation to CpG island (“Relation_to_UCSC_CpG_Island”), enhancer annotation (450 K enhancer), classified group by CMS_*k*_, and top 10,000 importance probes. Annotated genes, relation to CpG island, and enhancer annotation are followed by EPIC manifest. Colors of “UCSC_RefGene_Group” and “Relation_to_UCSC_CpG_Island” are shown in right panel. Upper left triangle and lower right triangle indicate the isolation pattern of the class. For example, red upper left triangle means high *β*-value is observed in most of group A sample and not observed in other groups. Details of annotation method of CMS_*k*_ are described in Materials and Methods. **(B)** *EDAR*. **(C)** *FAM13A*. **(D)** *PRDM8*

One characteristic example of the effect of *MYCN* amplification on DNA methylation is the *MYCN* gene locus methylation status (Fig. 4A). We focused on probes within 10 kb of the transcription start site (TSS),100 kb was the conservative threshold of regulation by enhancer [38]. We found hypomethylation of the enhancer regions in the *MYCN* locus, and among the 41 probes located within approximately ±100 kb of TSS, 27 probes were included in the top 10,000 *importance* probes and 15 out of the 27 probes were CMS-_*A,l*_ (red lower right triangle), which indicated hypomethylation in group A samples Ten probes were located on “450k_Enhancer,” and eight probes were classified as CMS_*A*_. These results emphasize the significance of the lack of DNA methylation in the *MYCN* genetic region, including its enhancer region. That high importance was consistent with SHAP method (Fig. S8) [39].

Group A-specific genes had noticeable changes in DNA methylation patterns in the enhancer region (Fig. 4B, C, and D, Table S4). *EDAR* had 25/36 CMS_*A*_ with 24 CMS_*A.u*_, and 6/25 probes were “450k_Enhancer” *FAM13A* had 5/16 CMS_*A*,*u*_, and 5/5 top *importance* probes, and 3/5 important probes were “450k_Enhancer” *PRDM8* has 24/88 CMS_*A*_ with 21 CMS_*A,u*_, 15/24 were top *importance* probes, and 5/15 probes were “450k_Enhancer” These results support the hypothesis that *MYCN* amplification is related to enhancer DNA methylation.

### Prognosis of patients using DNA methylation data

**Fig. 5:**
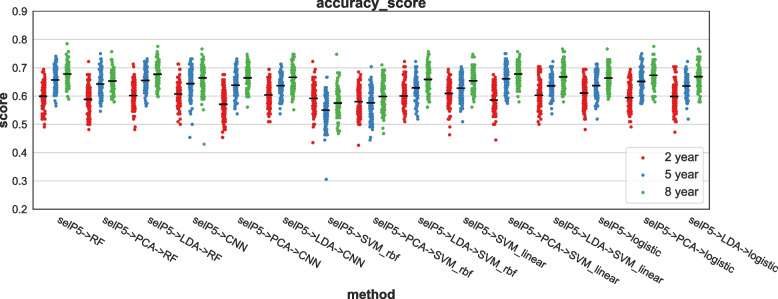
Prediction result of prognosis. Classification methods are listed on X-axis. Y-axis denotes accuracy score. Prediction was performed to 2-, 5-, and 8-year survival. selP5 means top 5% probes were selected using the select percentile method. PCA, principal component analysis; LDA, linear discriminant analysis; RF, random forest; CNN, convolution neural network; SVM_rbf, support vector machine with kernel type rbf; SVM_linear, support vector machine with kernel type linear; logistic, logistic regression

In this section, we tested the predictive power of the DNA methylation signature for patient prognosis. We used three kinds of survival information: outcomes (“censored” or “not censored” for the event including death of disease, progression or relapse) at two years, five years, and eight years after diagnosis. Outcomes at two years imply how aggressive the tumor character is, and survival probability at eight years after diagnosis nearly exhibits a plateau even with the *MYCN*-non-amplified cases. We tested five classification algorithms: RF, convolutional neural network (CNN), support vector machine with kernel type rbf (SVM_rbf), support vector machine with kernel type linear (SVM_linear), and logistic regression. The mean accuracy score across the algorithms was approximately 0.6 (Fig. 5). As expected, in all classifiers, 8 yr-EFS exhibited the highest accuracy score, and 2 yr-EFS exhibited the lowest. Because the survival status at eight years after diagnosis is close to the overall survival rate of the patients, DNA methylation signature may have the predictive power to closely determine each patient’s outcome. When probes were selected by selecting the percentile and data were dimension-decomposed by linear discriminant analysis (LDA), RF, logistic regression, and SVM_linear exhibited better scores. SVM_rbf and CNN exhibited larger variance, probably because of a lack of learning data (Table S5).

## Discussion

Comparative epigenomics in cancer challenging. A major limitation in large-scale epigenomics data analysis is data complexity, particularly compared to genome sequence data, in which variation patterns are well modeled. The application of machine learning models to DNA methylation data has progressed in recent years, including cancer state prediction and age prediction [40–45]. We used RF as an analytical model for DNA methylation data in this study. RF-based analysis successfully proposed a novel patient group of NB within pre-determined groups (D1 and D2 in Fig. 2). These groups showed distinct DNA methylation and prognosis patterns even when the age of onset variable was controlled (Fig. 3). This indicates that DNA methylation is a potential epigenetic marker to distinguish the intermediate-risk group of NB (D1) from within the low-risk group (D) and that RF is suitable for DNA methylation analysis.

Although we discussed using RF, which is a relatively simple decision-tree based ML algorithm, there are more sophisticated classifier; XGBoost [46], LightGBM [47] and extraTrees [48]. Table S6 showed the result of those classifier. Because of computation time, we used only the probes of top 10,000 variance of *β*-values. XGBoost and LightGBM showed better score for the group A and B despite of computation time. The LightGBM showed similar score to RF for all case. The extraTrees showed similar score for RF. Non tree-based models showed worse score. Because this pilot work aims to evaluate of applicability of ML method to DNA methylation data, we took advantage of computation efficiency of RF. It is possible to develop more sophisticated comparative DNA methylation analysis algorithm if a lot of computation resources and samples are available.

Our probe annotation comparison showed that probes in the enhancer region had strong classification power for *MYCN*-amplified tumors (Fig. 3; Figs. S4 and S5), confirming results from previously conducted DNA methylation analysis for NB [20, 21]. Although DNA methylation around the TSS and CGI regions is generally used as a tumor epigenetic marker, its classification power is still insufficient compared to that of the gene expression signature. This may be due to the low variance of *the β*-value (Table S2, var. of TSS and CGI). Variance of variables is an important factor for machine learning. This notion was supported by the positive correlation between the variance of *the β value* and *the obs/sim* ratio of the f1-score (Fig. 3C and D). In addition, “select percentile by ANOVA *p*-value” was a more cost-effective feature selection method than probe annotation (Fig. 3). Those result were consistent when prediction ability was measured in accuracy (Fig. S7). Our work provides basis for ML-based DNA methylation analysis and DNA methylation of enhancer region for classification of *MYCN*-amplified NBs.

To evaluate the difference at the DNA methylation site level, we proposed *an importance* and Gini-impurity-based analysis model (Fig. 4 and Figs. S4 and S5). This approach was useful for visualizing the direction of DNA methylation changes (Fig. 4). Although this method can provide intuitive results, the results are parameter sensitive (Fig. S4 and S5). This analysis suggests that changes in DNA methylation can be evaluated in specific genes. The most important NB prognostic marker, *MYCN* gene amplification, is accurate. We showed that *MYCN*-amplified samples (group A) were a distinct, characteristic group in the PCA of DNA methylation status (Fig. 1B). We also found that DNA methylation of enhancer regions was enriched in group A-related methylation probes, suggesting that *MYCN* amplification may play a role in dynamic changes in DNA methylation status in NBs, thereby leading the tumor cells to acquire an aggressive character (Fig. 4).

One of the limitations of this study is that our machine learning predicts known prognostic factors (such as tumor stage and age at diagnosis) which may sometimes contain certain deviations. Pure data-driven analysis may overcome this limitation if sufficient sample size is available.

Our approach provides new insight into the NB molecular data analysis. Previous reports demonstrated that DNA hypermethylation of promoter regions with CGI in some genes, such as *the protocadherin β* gene family and cytochrome p450 (CYP26C1), is also related to poor prognosis in patients with NB [17, 18]. Herein, CGI probes of these genes were included in the top 10,000 importance probes (rank 2564 and 5613, respectively), indicating that probe selection with importance/CMSk score is a useful method for identifying additional methylation markers for NB classification. Moreover, machine learning with RF enhanced the prognosis of NBs, particularly with high- and intermediate-risk types (Fig. 1). We identified some candidate genes (Fig. 4), including *FAM13A-AS,* which is an autophagy-related lnc-RNA [49]. Using their DEG analysis, we compared stages IV and IVs NBs and showed that the enhancer region was hypermethylated in group A and hypomethylated in group C, which is consistent with the previous report [49]. Accordingly, the *FAM13A-AS* lower expression was associated with worse prognosis for NB patients (Group A, Fig. S6a), *FAM13A* expression was higher in *MYCN*-amplified cells (Fig. S6b) of NB patients with poor prognosis (Fig. S6c, Analysis of KOCAK NB database in R2 database). *PRDM8* is an important gene in NB. However, in dyskeratosis congenita (DSK), hypermethylation was observed in the promoter region [50], and its pattern was similar to that of group A (Fig. 4). In addition, knockout of this gene impaired the neuronal differentiation of iPSCs. These results suggest that *PRDM8* may play an important role in NB progression [51]. In fact, lower expression of *PRDM8* was associated with poor prognosis in patients with NB (Fig. S6D, Analysis of SEQC NB database in R2 database). These results may contribute to future NB treatment.

## Conclusions

In conclusion, our analysis revealed that DNA methylome data can help to understand cancer molecular features, and machine learning is a powerful tool for analyzing cancer epigenome data. The advantage of machine learning is the use of data-driven analysis, which does not require a specific analysis model.

## Methods

All methods were carried out in accordance with relevant guidelines.

### Infinium HumanMethylation450 beadchip array data and generating *β*-value

We collected HumanMethylation450K BeadChip array (Illumina) dataset of patients with NB, with their clinical information from four different research projects. Raw idat files were obtained from the Gene Expression Omnibus (GEO) database (GSE715 [22] and GSE120650 [37]). For the target dataset, we obtained an idat file from the database (https://ocg.cancer.gov/programs/target/projects/neuroblastoma). Methylome data obtained from Japanese NB were provided by the Japan Children’s Cancer Group Neuroblastoma Committee (JCCG-JNBSG) collaborative work and will be published elsewhere (Ohira et al., in preparation).

To enumerate and normalize the methylation data, we used the Minfi package (v1.26.2) in R open-source statistical software (v3.5.3) [52]. Background correction and normalization were conducted using the *ProprocessIllumina* method [52]. *The β*-value was calculated as *β* = *M /* (*M* + *U* + 100), where *M* is the methylated value and *U* is the unmethylated value [53]. To annotate probes on the Illumina array, the manifestation of the EPIC array was referenced, which is enriched with enhancer information [52].

### Random forest settings

RF models were implemented using the *RandomForestClassifer* class of the scikit-learn v0.17 Python package. In all cases, the weight of the sample was controlled by *the class_weight* option.

In the case of Tables 1, 10% of the probes were selected by select percentile method using “chi2” option. The ranking of importance in Fig. 4 was calculated in this result. For inter-dataset comparison, we used the whole data for training and testing to calculate scores. For intra-dataset comparison, we selected 70% of the samples for the training dataset and 30% for the test data. *Max_depth* was set as four, and *n_estimator* was 10,000 for RF parameters; the number of replications was set to 100.

For feature selection analysis in Fig. 3, we set n_estimater as 10,000, and no limit was set for max_depth.

### XGBoost, LightGBM and extraTrees

The version of these classifiers were below: XGBoost (1.6.1, pypi_0) and LightGBM (3.2.1, py310he9d5cce_0) and scikit-learn for extraTrees (1.0.2, py310hc081a56_0).

### Survival time analysis

Survival data analysis was performed using the *Lifelines* package in Python [54]. To evaluate the survival effect of each probe, we calculated the log-rank *p*-value for each probe. The samples were separated by the mean *β*-value, and the *p*-value was collected using the Benjamini–Hochberg method.

### Definition of probe groups

All probe definitions in this study were based on the following Illumina manifest: “Promoter” referred to the probes that included any of TSS1500, TSS200, and 1stExon. “TSS” referred to the probes of TSS1500 and TSS200. The term “CGI” was used for “island” in the “Relation_to_UCSC_CpG_Island” column. “pha5enha” encompassed probes that were annotated in “Phantom5_Enhancers.” “450Kenha” referred to probes of TRUE in “450k_Enhancer.” “DMR,” “RDMR,” and “CDMR” were used for probes annotated in “DMR,” “RDMR,” and “CDMR” in the “DMR” column, where DMR refers to a differentially methylated region. RDMR refers to reprogramming-specific differentially methylated region. CDMR refers to tje cancer-specific, differentially methylated region. “DNase” included probes annotated in “DNase_Hypersensitivity_NAME.” “TFBS” referred to probes annotated in “TFBS_NAME.” “OpenChr” referred to probes annotated in “OpenChromatin_NAME.” and finally, “SNP” referred to probes of TRUE in “SNP_ID.”

### Classification power for genes

To evaluate the classification power for the single-probe level, we introduced the CMS_*k*_ index, which is based on Gini-impurity. First, we set *G*(*r*) as a Gini-impurity for a *β*-value, *r*. We used the equation

$$G(r)=1-\sum_{k=1}^K{p}_{k,u}(r)\ {p}_{k,l}(r)$$

where *p*_*k*, *u*_(*r*) and *p*_*k*, *l*_(*r*) are the fractions of class *k* ∈(A,B,C,D) with the upper and lower values of *r*. *r*_0_ is the *r* value when *G*(*r*) is at the least value.

$${r}_0=\underset{r\in \left\{0,1\right\}}{\arg\ \min }G(r)$$

We calculated *r*_0_ for each probe across all samples.

Next, we evaluated the degree of classification at the point of *r*_0_. We defined *p*_*k*, *u*_(*r*) and *p*_*k*, *l*_(*r*) as the proportion of group *k* (∈(A,B,C,D)) samples upper and lower *β values* than *r*, respectively. r_0 was calculated in RF result of max depth was 2.

If *p*_*k*, *u*_(*r*) was over a threshold (*θ*), we define those probes as CMS_*k*_. In the case of cg12343591 (Fig. 5), *r*_*0*_ = 0.767339, and *p*_*k*, *u*_(*r*) and *p*_*k*, *l*_(*r*) were below: { *p*_*A*, *u*_(*r*) = 0.163, *p*_*B*, *u*_(*r*) = 0.918, *p*_*C*, *u*_(*r*) = 0.982, *p*_*D*, *u*_(*r*) = 0.927} vs { *p*_*A*, *l*_(*r*) = 0.836, *p*_*B*, *l*_(*r*) = 0.082, *p*_*C*, *l*_(*r*) = 0.018, *p*_*D*, *l*_(*r*) = 0.073}. In this case, cg12343591 was CMS_*A,u*_ when θ = 0.8 was chosen, but not CMS_A_ when *θ* = 0.9. The effect of *θ* on the CMS_*k*_ is shown in Fig. S4. In this study, we chose *θ* = 0.8 as our threshold.

### Patient prognosis

We used two classes of outcomes: no event or event, which represented death from disease and relapse. Five percent of probes were chosen using the select-percentile method. Data pre-processing was performed using PCA or LDA. The dimensions of the PCA were tuned by a grid search and fixed at 2. Parameter tuning was performed using the RandomizedSearchCVfunction in scikit-learn. For RF, max_depth = 3,4,5,6 *n*_estimators were fixed at 1000. For the logistic regression, *C* = [0,100). Solver = lbgf, multi_class = auto, max_iter = 1000. For the SVM, the kernel functions of “linear” and “rbf” were compared in independent classifiers, “SVM_linear” and “SVM_rbf,” respectively. The tuned parameters were the same, and C = [0,100), gamma = [0,1). For the CNN, hidden_layer_sizes: [(50,),(50,50),(100,),(100,100),(100,100,100),(200,),(200,200)], max_iter:[100,500,1000], and batch_size: [10] were used.

### Hyper parameter of classifier model comparison

We used the probes with top 10,000 variance of *β*-value. For tree-based classifier, for time efficiency, we fixed *max_depth* as 5, and 10% of probes were selected by select percentile. For non tree-based classifier which overfitting does matter, we used “grid search” algorithm to seek the best parameter of select percentile, with the range as [1, 3, 5, 7, 9]. In addition, other parameter tuning was performed. For logistic regression, C = [0,1). For SVM and CNN, the parameter was same as the section, “Patient prognosis”.

## Supplementary Information

**Additional file 1: Fig. S1. **PCA result for sample categories. PC1–4 were colored by each classification scheme. Marker information were listed in each Fig. A) Data source. B) INSS stage. C) MYCN amplification status. D) Age at diagnosis. **Fig. S2 **Intra-dataset prediction ability. Training dataset were listed on y-axis and test data was listed on x-axis. (A) Precision. (B) recall. **Fig. S3 **Relationship between Δβ|D1-D2| and log rank test’s FDR (LRFDR). (A) Δβ|D1-D2| was calculated between D1and D2. (B) Δβ|D1-D2| was calculated between B1 and D2. (C) Δβ|D1-D2| was calculated between B1 and D2. Purple marker indicated Δβ|D1-D2| > 0.3 and LRFDR < 0.01. **Fig. S4 **Distribution of probe annotation groups in binned importance. x-axis is the ranked probes by importance. y-axis is the proportion of probes in the focal bin. Probe annotation group was colored. **Fig. S5 **Relationship between rank by importance and number of classifying probes. A) x-axis is the ranked probes by importance. y-axis is the number of classifying probes. Line color illustrated each θ. Top row showed the number of CMSk. In the middle and bottom rows, it was shown the number of CMSk with upper and lower than 𝑟!, respectively. Column label indicated focal class. **Fig. S6 **A) Survival time analysis of 476 NB patients divided by gene expression level of FAM13A-AS. Gene expression data and survival time data were obtained from the R2 database (https://hgserver1.amc.nl/). Kocak data were selected. “Scan” option was selected for sample grouping. B) Gene expression level of FAM13A for the data of Henrich et al. [22]. Box plot of FAM13A gene expression in MYCN-amplified (*n* = 33) and non-amplified (*n* = 72) tumors. C) Kaplan–Meier survival curves of 476 patients with NB divided by FAM13A expression, with same setting of Fig. S6A. D) Kaplan–Meier survival curves of 498 patients with NB divided by PRDM8 expression with same setting of Fig. S6A. Gene expression data were obtained from the SEQC data. **Fig. S7 **Remake Fig. 3 for accuracy as index.** Fig. S8 **Probe contribution of classification measuring by SHAP value. **Table S1.** Number of samples used in this study.** Table S2. **Prediction ability of each probe annotation for group A and B, **Table S5.** Mean accuracy and its standard deviation,** Table S6. **Classification ability was compared.

**Additional file 2.**

**Additional file 3.**

## Data Availability

All the data and materials are presented in this manuscript and in the Supplementary File.

## Abbreviations

NB: Neuroblastoma
RF: Random forest
INSS: International Neuroblastoma Staging System
INRG: International Neuroblastoma Risk Group
CIMP: CpG island methylation phenotype
ML: Machine learning
SVM: Support vector machine
LRFDR: Log-rank test’s false discovery rate
CNN: Convolutional neural network
GEO: Gene Expression Omnibus
