# Supplementary material for: Comparative epigenomics by machine learning approach for neuroblastoma
Source: BMC Genomics. 2022 Dec 27;23:852. doi: 10.1186/s12864-022-09061-y (PMC9793522; doi:10.1186/s12864-022-09061-y)
Supplement: Supplementary file 1 — Additional file 1: Fig. S1. PCA result for sample categories. PC1–4 were colored by each classification scheme. Marker information were listed in each Fig. A) Data source. B) INSS stage. C) MYCN amplification status. D) Age at diagnosis. Fig. S2 Intra-dataset prediction ability. Training dataset were listed on y-axis and test data was listed on x-axis. (A) Precision. (B) recall. Fig. S3 Relationship between Δβ|D1-D2| and log rank test’s FDR (LRFDR). (A) Δβ|D1-D2| was calculated between D1and D2. (B) Δβ|D1-D2| was calculated between B1 and D2. (C) Δβ|D1-D2| was calculated between B1 and D2. Purple marker indicated Δβ|D1-D2| > 0.3 and LRFDR < 0.01. Fig. S4 Distribution of probe annotation groups in binned importance. x-axis is the ranked probes by importance. y-axis is the proportion of probes in the focal bin. Probe annotation group was colored. Fig. S5 Relationship between rank by importance and number of classifying probes. A) x-axis is the ranked probes by importance. y-axis is the number of classifying probes. Line color illustrated each θ. Top row showed the number of CMSk. In the middle and bottom rows, it was shown the number of CMSk with upper and lower than 𝑟!, respectively. Column label indicated focal class. Fig. S6 A) Survival time analysis of 476 NB patients divided by gene expression level of FAM13A-AS. Gene expression data and survival time data were obtained from the R2 database (https://hgserver1.amc.nl/). Kocak data were selected. “Scan” option was selected for sample grouping. B) Gene expression level of FAM13A for the data of Henrich et al. [22]. Box plot of FAM13A gene expression in MYCN-amplified (n = 33) and non-amplified (n = 72) tumors. C) Kaplan–Meier survival curves of 476 patients with NB divided by FAM13A expression, with same setting of Fig. S6A. D) Kaplan–Meier survival curves of 498 patients with NB divided by PRDM8 expression with same setting of Fig. S6A. Gene expression data were obtained from the SEQC data. Fig. S7 Remake Fig. 3 fo [file 12864_2022_9061_MOESM1_ESM.pdf]

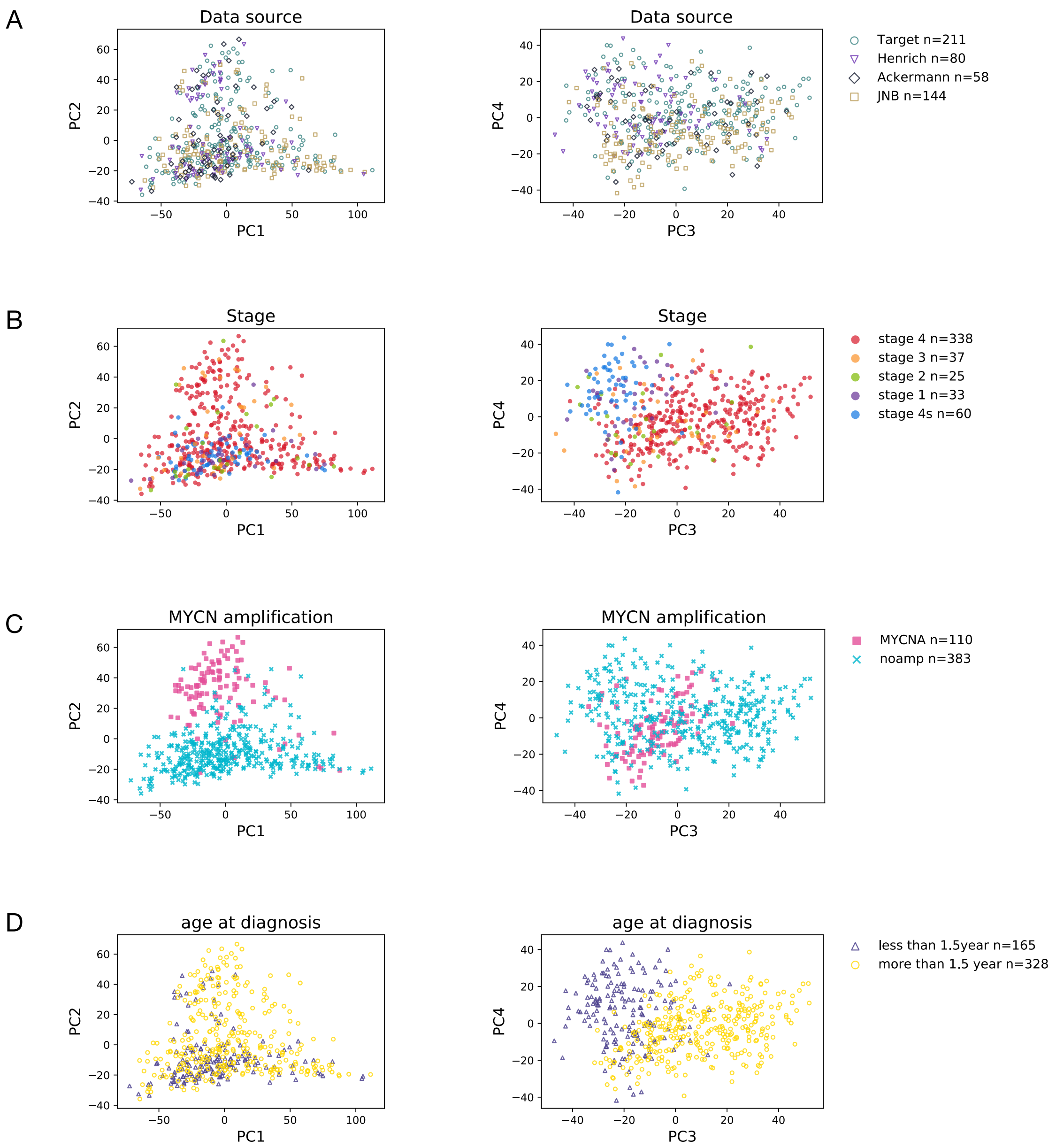

Fig. S1 PCA result for sample categories. PC1-4 were colored by each classification scheme. Marker information were listed in each figure. A) Data source. B) INSS stage. C) *MYCN* amplification status. D) Age at diagnosis.

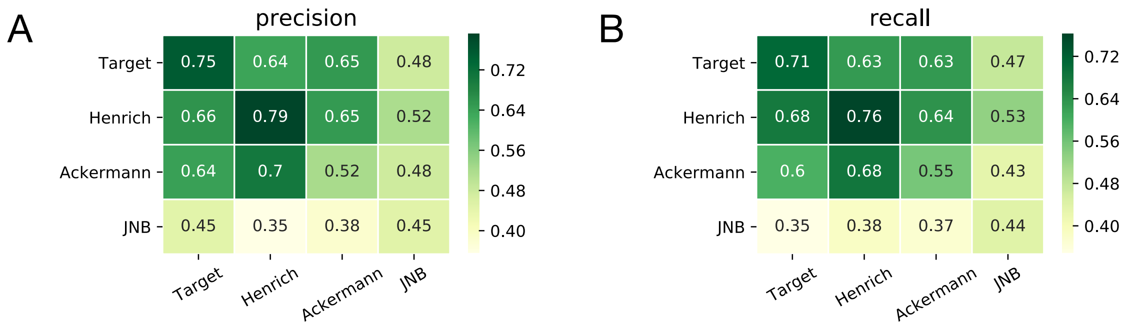

Fig. S2 Intra-dataset prediction ability. Training dataset were listed on y-axis and test data was listed on x-axis. (A) Precision. (B) recall.

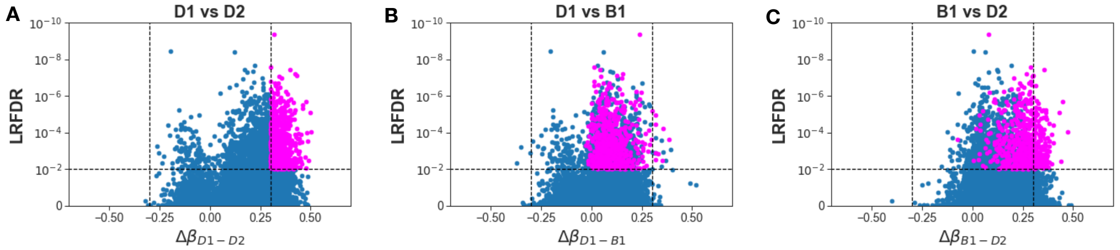

Fig. S3 Relationship between $\Delta\beta_{|D1\text{-}D2|}$ and log rank test's FDR (LRFDR). (A) $\Delta\beta_{|D1\text{-}D2|}$ was calculated between D1and D2. **(B)** $\Delta\beta_{|D1\text{-}D2|}$ was calculated between B1 and D2. **(C)** $\Delta\beta_{|D1\text{-}D2|}$ was calculated between B1 and D2. Purple marker indicated $\Delta\beta_{|D1\text{-}D2|}$>0.3 and LRFDR < 0.01.

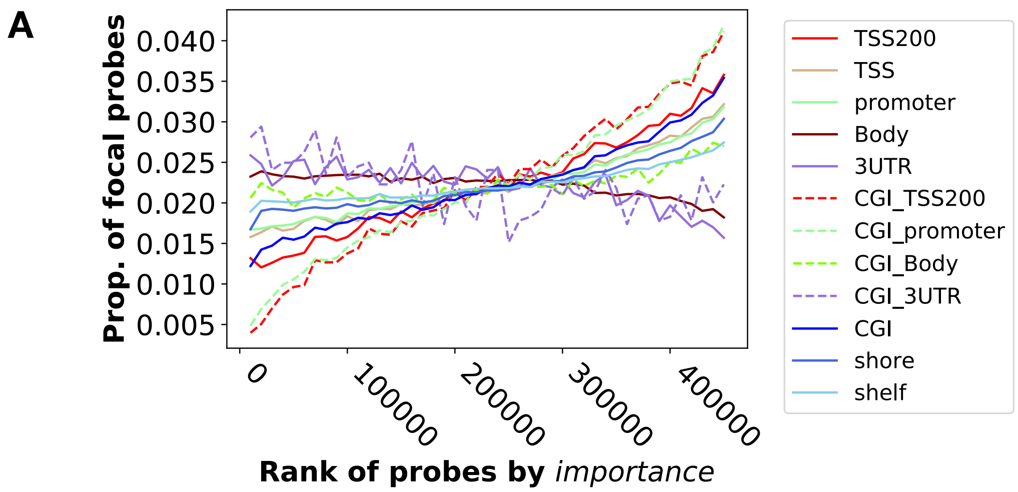

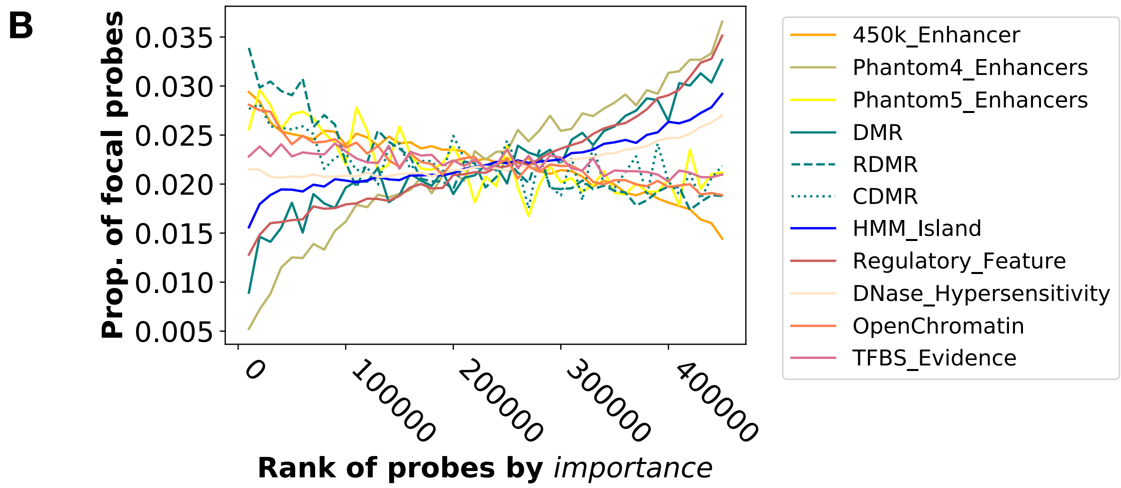

Fig. S4 Distribution of probe annotation groups in binned *importance*. x-axis is the ranked probes by *importance*. y-axis is the proportion of probes in the focal bin. Probe annotation group was colored.

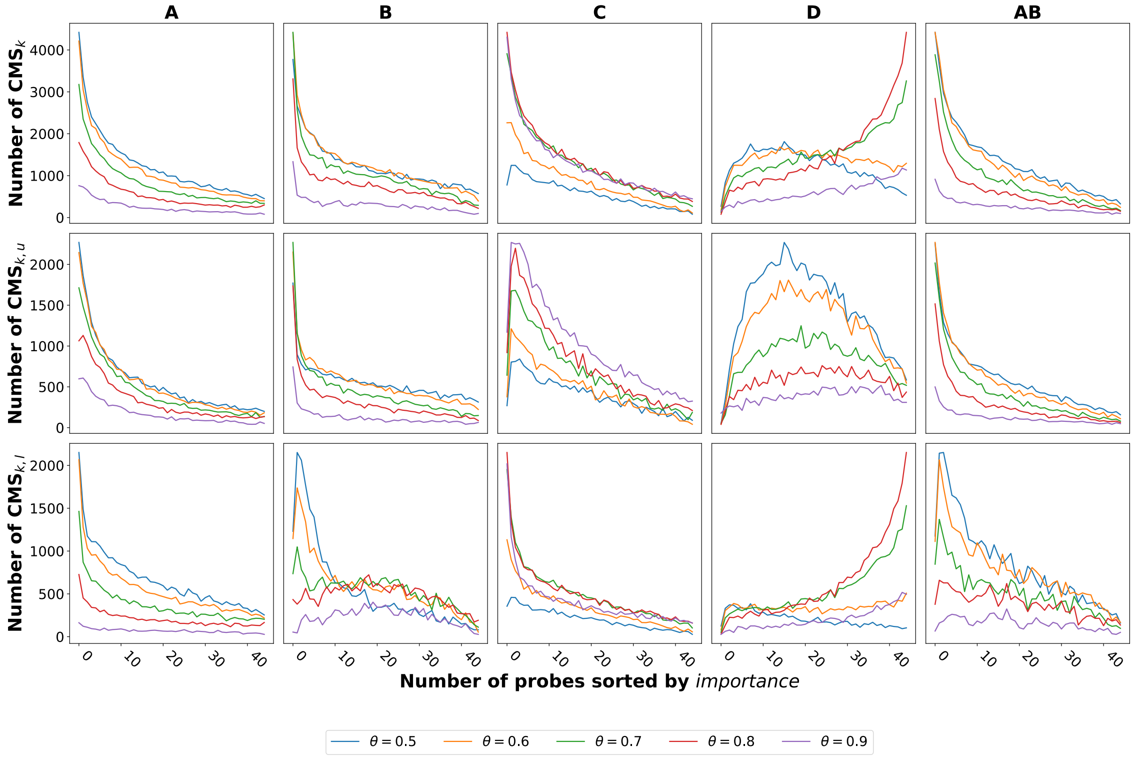

Fig. S5 Relationship between rank by *importance* and number of classifying probes. A) x-axis is the ranked probes by *importance*. y-axis is the number of classifying probes. Line color illustrated each θ. Top row showed the number of CMS$_k$. In the middle and bottom rows, it was shown the number of CMS$_k$ with upper and lower than r_0, respectively. Column label indicated focal class.

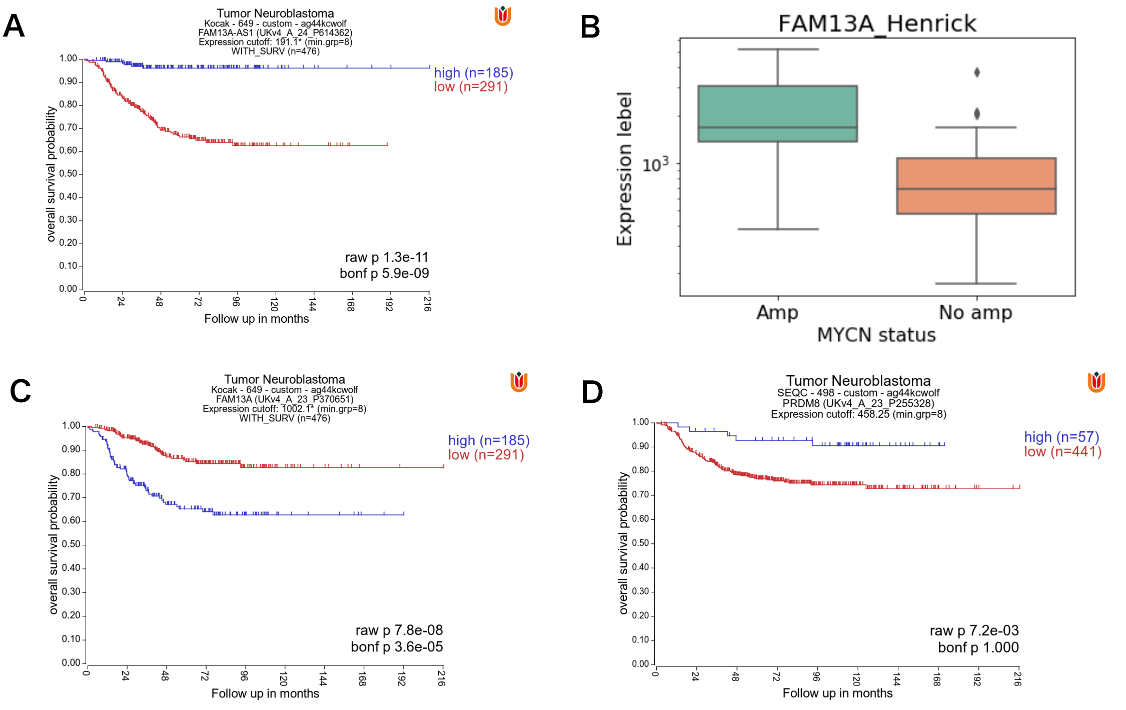

Fig. S6 A) Survival time analysis of 476 NB patients divided by gene expression level of *FAM13A-AS*. Gene expression data and survival time data were obtained from the R2 database (https://hgserver1.amc.nl/). Kocak data were selected. “Scan” option was selected for sample grouping. B) Gene expression level of *FAM13A* for the data of Henrich *et al.* [22]. Box plot of FAM13A gene expression in MYCN-amplified (n = 33) and non-amplified (n = 72) tumors. C) Kaplan–Meier survival curves of 476 patients with NB divided by *FAM13A* expression, with same setting of Fig. S6A. D) Kaplan–Meier survival curves of 498 patients with NB divided by *PRDM8* expression with same setting of Fig. S6A. Gene expression data were obtained from the SEQC data.

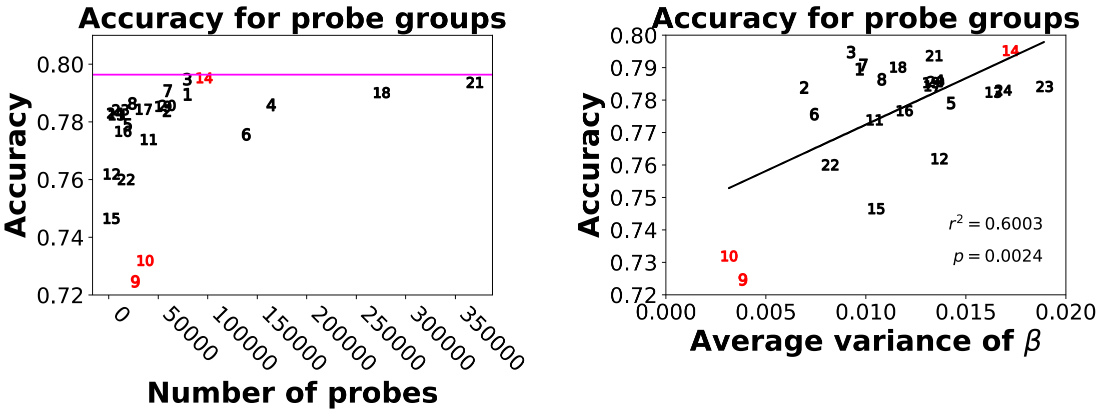

Fig. S7 Remake fig 3 for accuracy as index.

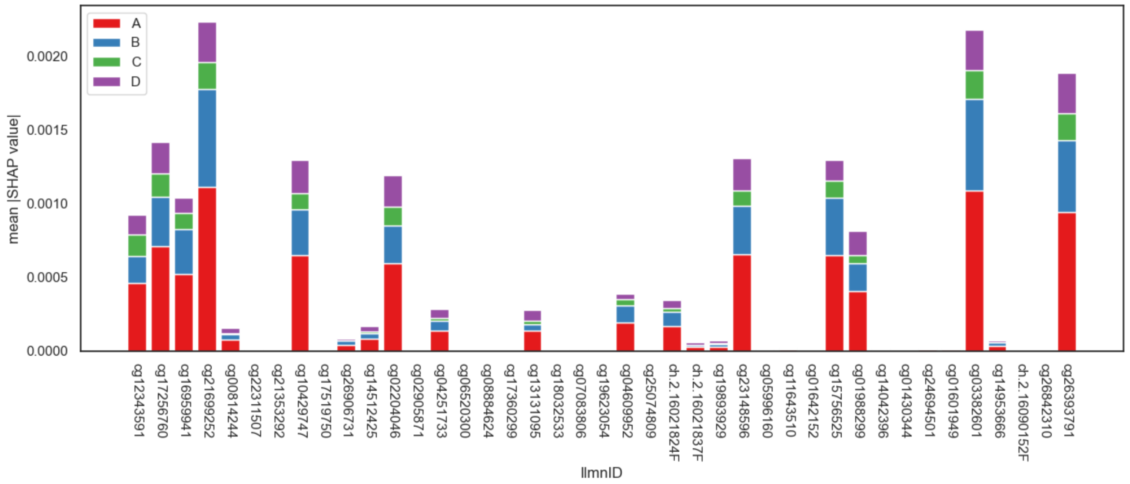

Fig. S8 Probe contribution of classification measuring by SHAP value.

Dataset	Target	Henrich	Ackerman	JNB	total
**INSS stage**					
4	165	41	29	103	338
3	6	8	5	18	37
2	1	8	9	7	25
1	15	8	7	3	33
4s	24	15	8	13	60
Total	211	80	58	144	493
**MYCN amplification**					
Y	50	25	15	20	110
N	161	55	43	124	383
Total	211	80	58	144	493

Table S1. Number of samples used in this study.

Probe annotation	probe_num	A_f1	B_f1	var
promoter	78798	0.8955	0.8650	0.0096
TSS200	58151	0.8724	0.8597	0.0068
TSS	78798	0.8917	0.8682	0.0092
Body	163726	0.8912	0.8670	0.0136
3UTR	18534	0.8692	0.8576	0.0142
CGI	138386	0.8867	0.8528	0.0073
shore	58921	0.9021	0.8660	0.0098
shelf	23349	0.8839	0.8666	0.0107
CGI_promoter	26416	0.8337	0.8018	0.0038
CGI_TSS200	36313	0.8545	0.8065	0.0031
CGI_Body	39719	0.8688	0.8574	0.0104
CGI_3UTR	2259	0.8724	0.8409	0.0136
pha5enha	7269	0.8887	0.8584	0.0163
450Kenha	95986	0.8983	0.8698	0.0172
pha5enha_CGI	2070	0.8862	0.8219	0.0104
450Kenha_CGI	14017	0.8978	0.8489	0.0119
DMR	34643	0.8992	0.8551	0.0132
DNase	275166	0.8928	0.8664	0.0115
TFBS	54338	0.8837	0.8653	0.0132
OpenChr	58724	0.8778	0.8661	0.0134
SNP	369310	0.8985	0.8749	0.0133

Table S2. Prediction ability of each probe annotation for group A and B

	2yrs	5yrs	8yrs
selP5>RF	0.609±0.044	0.657±0.037	0.678±0.037
selP5>PCA>RF	0.591±0.041	0.643±0.041	0.653±0.035
selP5>LDA>RF	0.613±0.045	0.655±0.037	0.677±0.036
selP5>CNN	0.61±0.046	0.644±0.051	0.665±0.049
selP5>PCA>CNN	0.602±0.04	0.638±0.041	0.664±0.037
selP5>LDA>CNN	0.607±0.039	0.637±0.036	0.666±0.04
selP5>SVM_rbf	0.6±0.036	0.55±0.053	0.575±0.048
selP5>PCA>SVM_rbf	0.585±0.042	0.576±0.049	0.599±0.05
selP5>LDA>SVM_rbf	0.61±0.037	0.629±0.038	0.659±0.041
selP5>SVM_linear	0.618±0.042	0.628±0.04	0.654±0.039
selP5>PCA>SVM_linear	0.607±0.038	0.661±0.038	0.678±0.032
selP5>LDA>SVM_linear	0.612±0.036	0.636±0.035	0.668±0.038
selP5>logistic	0.621±0.038	0.637±0.039	0.664±0.035
selP5>PCA>logistic	0.604±0.038	0.652±0.04	0.673±0.036
selP5>LDA>logistic	0.611±0.036	0.636±0.034	0.669±0.038

Table S5. Mean accuracy and its standard deviation

	RF	XGB	LGBM	exTrees	logreg	CNN	SVM_rbf	SVM_linear
mean_score	0.802	0.810	0.814	0.794	0.714	0.664	0.703	0.693
A_precision	0.932	0.963	0.971	0.932	0.911	0.893	0.905	0.872
B_precision	0.838	0.847	0.844	0.811	0.836	0.820	0.829	0.850
C_precision	0.625	0.626	0.642	0.603	0.612	0.582	0.587	0.563
D_precision	0.577	0.556	0.577	0.644	0.563	0.465	0.578	0.504
A_recall	0.894	0.939	0.943	0.877	0.897	0.879	0.893	0.895
B_recall	0.925	0.929	0.929	0.954	0.900	0.889	0.900	0.851
C_recall	0.654	0.613	0.664	0.584	0.667	0.580	0.596	0.589
D_recall	0.403	0.414	0.417	0.356	0.411	0.355	0.420	0.465
A_f1	0.911	0.950	0.956	0.902	0.902	0.882	0.897	0.881
B_f1	0.879	0.885	0.884	0.876	0.866	0.849	0.862	0.849
C_f1	0.630	0.606	0.642	0.576	0.624	0.547	0.579	0.564
D_f1	0.467	0.464	0.474	0.445	0.465	0.377	0.474	0.475

Table S6. Classification ability was compared
